# The Foreign Journals: Pathology, Practical Medicine, and Therapeutics

**Published:** 1840-01

**Authors:** 


### PATHOLOGY, PRACTICAL MEDICINE, AND THERAPEUTICS.

### *Lithotripsy performed by the water of the Royal Fountain of Recoaro. Memoir presented to the Society of Sciences in Modena.* By Professor Brera.

This memoir contains three cases of the lithotriptic powers of the above waters, not in producing the dissolution of the calculi, but such a weakening of their cohesion, that they passed off with the urine either in fragments or in the form of fine sand. The quantity of water taken was two pints every morning, and it was continued for two or three months, or as long as the attack lasted, where there was no perfect calculus formed. In two cases there was chronic inflammation of the bladder, together with a loss of its contractile power in the third, from which the patients perfectly recovered by the use of the water. The calculi were of the lithic acid kind in each case. No analysis of the water is given, but the following analysis by Peregrini, of the water of St. Catherine's well in the Valtelline, is said to resemble that of Recoaro:

Besides much carbonic acid it contains

| | |
|---|---|
| Sulphate of soda - - - - | 50·85 |
| Chloride of sodium - - - - | 28·63 |
| Sulphate of magnesia - - - | 9·83 |
| Carbonate of do. - - - - | 53·17 |
| Bicarbonate of zinc - - - | 778·89 |
| Silicate of alumina - - - - | 36·06 |
| Carbonate of iron - - - - | 277·03 |

The quantity of water analysed is not mentioned.—*Omodei, Annali,* vol. lxxxvi.

---

*Remarkable Tendency to Hemorrhage in a family.* By Dr. DU BOIS, of Neuchâtel, Switzerland.

THIS affection, which consists of an extreme fluidity of the blood, or a weakness of the capillary vessels, which are ruptured by the slightest violence, and have but little contractile power, is not uncommon in the west of Germany, and in the Rhenish provinces. It is hereditary in a striking degree; though only males are affected by it, to whom it is frequently transmitted by their mother, who is free from its influence. In some families scarcely a single male arrives at maturity, from this cause; and the person thus diseased bears the significant name of *Bluter* or Bleeder.

A robust gardener of Neuchâtel married a stout, healthy woman from Nassau, in whose family, according to her account, no Bluter had been known. By her he had a family, consisting of five boys, and one girl who never exhibited any symptoms of this complaint, and died in convulsions when three years of age. Of the five boys, one died of convulsions on the day of his birth; three died of hemorrhage; and the last, now seven years old, will probably soon follow his brothers, from the same cause. The symptoms exhibited the following course in all. A fortnight after birth, which was natural, ecchymoses began to appear in different parts of their bodies, spontaneously, or from the slightest pressure, and slowly disappeared, leaving a yellow tint behind them. About the end of the first year, but especially after the third, they were seized with violent epistaxis. The slightest puncture caused a great loss of blood; coughing produced hæmoptysis, and diarrhœa bloody stools in clots. The fourth bit his tongue at play, and died in a few days from the hemorrhage, which it was impossible to restrain. All were subject to frequent attacks of pain and swelling, with ecchymosis of the wrist, ankles, and knee-joints, attended with fever; the complaint usually lasted about a fortnight, and then disappeared with the subsidence of the swelling and removal of the ecchymosis. On one occasion, two leeches were incautiously applied to the knee of the eldest, the bites of which continued to bleed for three days, and were only stopped by the twisted suture. Except varicella, which the survivor has had, none of the others were attacked by infantile diseases, though prevalent in the neighbourhood. Dentition took place pretty early, and in a healthy manner. A tendency to diarrhœa, that was followed by bloody evacuations, was the sole affection of the organs of digestion to which they were liable. Their complexions were fair, with clear blue or brown eyes, and a skin remarkably fine and white. Their gums were always firm, and they never had ulcerations of the mouth or skin. Their intelligence was quite conformable to their age. Their urine was usually clear and limpid, but they had great tendency to perspire. The eldest died of epistaxis, at the age of twelve; the second died at the age of eight, of hemorrhage from all the mucous surfaces; and the third, as before mentioned, from biting his tongue, when twenty months old. The blood from these hemorrhages was very fluid, of the ordinary colour, and coagulated like other blood.

The surviving boy has undergone the same complaints as his brothers ; he is seven years old, of ordinary stature, delicate, rather thin, light complexioned, with light brown eyes, that are quick and intelligent; his skin is extremely white and transparent, with very little appearance of veins, that are very small, even on his hands and forearms; his face is exceedingly pale, but his nose, like that of his brothers, is of a bright red. His pulse is eight or ten beats quicker than in boys of his age, his breathing is normal, his gums are firm and sound, and the ends of his fingers exhibit no peculiarity. Respiration is puerile; there is slight hypertrophy, and the beats of the heart are very strong and smart (secchi), a circumstance also observed by his mother in the other children. The sounds of the heart are natural; and none of the children were subject to palpitation nor dyspnœa. No enlargement of the liver or spleen can be detected.

*Omodei, Annali,* vol. lxxxv., 1838.

---

*Singular Case of Absorption of Milk.* By Dr. D. Rasi.

A young lady of sound constitution married at fifteen, and removed from a damp and impure atmosphere to one remarkable for its freshness and purity. Not thirty days elapsed, before she had various nervous and uterine symptoms which indicated pregnancy. At the seventh month she had a severe pneumonia, with aggravation of the previous symptoms. The convalescence from this disease had scarcely commenced, when she was delivered of a healthy and mature child. Her health was for some time afterwards very delicate, and was only improved by a temporary removal to her native air. On her return thence, she again became pregnant, and had again the same nervousness and debility; but at the full period she bore another healthy child, which, though very weak, she was anxious to suckle. She was prevented, however, by a singular circumstance. Shortly after her delivery she had a severe fever, during which her breasts became very large and hard ; the nipples were swollen and firm, and there was evidently an abundant secretion of milk ; but neither the sucking of the infant, nor any artificial means could draw a singledrop of milk from the swollen glands.

It was clear that the lactiferous ducts were closed, and as the breasts continued to grow larger and more painful, purgatives and other means were employed to check the secretion of milk. After three days, the fever diminished somewhat, and was replaced by a constant cough, which was at first dry, but soon after was followed by the expectoration of simple mucus. After this, the cough diminished in severity, and the expectoration became very easy; but the sputa were no longer mucous, but composed of a liquid which had all the physical characters of genuine milk. This continued for fifteen days, the quantity of milk expectorated amounting to three ounces or more in the twenty-four hours. The breasts gradually diminished in size, and by the time the expectoration ceased, they had regained their natural dimensions.

The same complete obstacle to the flow of milk from the nipples recurred after the births of four children successively, and after that of the sixth she had similar symptoms of fever, but this time they were not followed by bronchitis or the expectoration of milk. She had in their stead copious sweatings, which with other severe symptoms reduced her to a cachectic state, and terminated fatally in a fortnight. *Bulletino delle Scienze Mediche, Aprile,* 1839.

---

*Case in which a Salivary Calculus was Developed in the Submaxillary Duct, or Canal of Wharton.* By M. Maisonneuve, Doctor in Surgery.

This case is interesting, on account of the infrequency with which earthy concretions are met with in the salivary ducts. Not above a dozen cases are on record, which are principally dispersed through the foreign journals.

The subject of the present case, a medical man, perceived in the month of June, 1839, a degree of painful swelling in the region of the right submaxillary gland, which diminished or disappeared at one time, and increased again at others, it being particularly troublesome when eating. This led the patient to expect that there was some obstruction impeding the flow of saliva (which is secreted in large quantities during mastication) through the submaxillary duct. In the course of the following month, a small tumour appeared under the tongue on the right side, which continued much of the same size till the month of September, the retention of saliva up to the time becoming greater and more troublesome. After every meal, the submaxillary gland was generally swelled, and could be plainly felt of an unusual size through the integuments. Pressure on the gland was painful, and, if exerted to a considerable degree, forced out a large quantity of thickened saliva into the mouth, mixed with white flocculi.

At this period the patient consulted M. Maisonneuve, who on examination discovered a small oblong tumour, about the size of an almond, beneath the tongue, on the right side of the frenum. On finding the orifice of Wharton's duct, and passing into it a small probe, a quantity of thickened and viscid mucus escaped, and on carrying the instrument a little further, it grated against a rough body, which was immediately suspected to be a calculus, and which M. Maisonneuve extracted, after considerable difficulty, the rough surface of the stone adhering closely to the walls of the duct. After the removal of the foreign body the unpleasant symptoms quickly disappeared, and the patient rapidly recovered. The calculus was of a dull white colour, and covered on its surface with a number of red points; it was of the shape and about double the size of a grain of barley, and rough and unequal. Its chemical composition was not ascertained.

[Cases of this kind, as the author has remarked at the commencement of the paper, are not at all common, and therefore they are worth recording; but the principal point of interest is wanting in the foregoing observation, namely, the chemical constitution of the calculus. In animals, as the ass and horse, in which salivary concretions are more common than in man, the composition of these calculi has been found principally to consist of carbonate of lime, mixed with phosphate of lime and animal matter, and sometimes carbonate of magnesia, none of which salts appear to be natural ingredients in saliva. A few years ago we ourselves met with a case in which several small calculi were developed either in the duct of the lachrymal gland, or in the folds of the conjunctiva at the upper and outer part of the eye, from which situation they escaped with considerable pain; their chemical composition was the same as that which seems to be most general in salivary calculi, only the phosphate of lime rather predominated over the carbonate. The causes which lead to the formation of the concretions are totally unknown.] *Gazette Médicale, Sept.* 1839.

---

### *On the Influence of Mercurial Preparations in Smallpox and Cowpox.* By Dr. BRIQUET.

THE author of this paper has shown in a preceding memoir (*see British and Foreign Medical Review.* Vol. VII., p. 552,) that mercurial plasters and ointment have a great effect in modifying the characters of variolous eruptions, in favorably influencing the progress of smallpox generally, and in preventing deformity from cicatrices. In the present article M. Briquet has endeavoured to ascertain in what manner mercurial preparations exert this favorable influence; and by means of performing a number of experiments, he has been enabled to establish the following facts:

1. Mercurial preparations, applied to the skin previously to the application of blisters or other irritating preparations, do not in any manner diminish their power of irritating the skin.

2. Mercurial preparations, if mixed with irritating substances, and applied together with them to the skin, in no way interfere with the action of the latter.

3. Mercurial plasters, if applied in various inflammatory eruptive diseases, as erysipelas, shingles, eczema, acne, furuncles, &c., very seldom if ever produce any diminution of inflammation, or in fact any effect whatever.

4. When applied after vaccination, preparations of mercury exert a marked influence over the course of cowpox. The vaccine virus frequently produces no effect at all; at other times a very small pustule forms, or only a simple vesicle filled with a whitish fluid and occasionally only a simple pimple or tubercle is developed. Thus the action of mercury in smallpox and cowpox seems to be identical. Mercury applied to the vaccine eruption after the fourth day has no influence over its course; and our author has shown in the preceding paper, that in smallpox, "in order that the plasters may have the effect of modifying the eruption, they must be applied before it has become pustular."

M. Briquet draws the conclusion from these facts, that mercurial preparations have no effect in simple inflammation of the skin, but only over some peculiar specific forms, in which the mercury does not exert its influence over the inflammation, but the virus itself, which it renders less active, or even inert. He asks, whether it is not probable that mercury taken into the system during the latent period between the reception of variolous infection and its development may not have the effect of preventing the disease altogether, or of greatly diminishing its intensity? He seems even to advise the administration of mercury to persons who have never been vaccinated, nor had smallpox during the prevalence of an epidemic of the latter disease. [One interesting result of these experiments is, that they afford an additional argument in favour of the idea that smallpox and cowpox are only modifications of the same disease.]

*Gazette Médicale*, 12*th Oct.*, 1839.

---

*Case of Ileus cured by Injection of Air.*

A CURASSIER, who suffered occasionally from colic, had a very severe attack in the beginning of August, 1838, in consequence of having eaten very freely of raw bacon, and afterwards drinking cold water. Vomiting ensued, but without relief to the pains, which continued to return in the umbilical region with considerable violence. Symptoms of decided enteritis followed; the vomiting became more severe and fecal; and the obstinate constipation of the bowels could not be overcome, even by the administration of pure mercury. In this state a quantity of air was thrown into the large intestines, and copious evacuation of the bowels followed with instant relief of all the symptoms, the constipation having lasted for eleven days.

*Medicinische Zeitung*, No. xxx. 1839.

---

*Disease of the Cæcum; Ileus and Gastrotomy.* By M. MONOD.

A WOMAN, aged twenty-five, habitually in good health, only she complained occasionally of pain at the epigastrium. These pains, at length, were accompanied by vomiting; notwithstanding, her general health was good. About a year ago she was hurt about the ileo-cæcal region, and afterwards the pains were increased: since (March, 1838,) they have been felt towards the hypogastrium. No vomiting; but colic and diarrhœa. From the third day of the last attack, a large and hard tumour appeared near the ileo-cæcal region. Instead of diarrhœa, constipation and vomiting of a greenish and transparent fluid; with progressive emaciation and paleness. The tumour had an ovoid figure, about four inches long and two to three across; it was hard, elastic, scarcely sensible to pressure. Examinations, per vaginam et rectum, disclosed nothing unnatural, except a hardness towards the right side of the pelvis.

M. Monod, after due consideration, resolved to have recourse to gastrotomy. He first made an incision about three inches over the tumour, from above downwards, and from without inwards, and penetrated by degrees as far as the peritoneum; this being cut, a fold of the intestine presented itself, which being recognized by its band of longitudinal fibres to be a portion of the colon, was replaced. M. Monod then brought out by his finger a fold of the small intestine, which was red and swelled, but not remarkably sensible; into this he made an incision, with scissors, about an inch and a half; immediately a considerable quantity of fecal matter escaped, with sensible relief to the patient; a thread was then passed through the mesentery, which was secured by strips of plaster, and the dressing completed with cerated lint and charpie. Two days after the patient died. *Dissection.* Inflamed peritoneum; sero-purulent fluid in its cavity; the intestinal folds within the pelvis covered with half concrete pus. The incision was made in the ileon about eight or nine inches from the cæcum; the cæcum very adherent behind; the superior folds of the small intestine above the artificial anus are a little dilated. The obstacle to the exit of the fæces was found in the superior and posterior part of the cæcum, at its junction with the ascending colon. The cæcum there presented a considerable contraction, so that only a female sound could be introduced; the little finger was found too large. At this place the cæcum, everywhere adherent to its surrounding parts, was connected to a scirrhous mass, about the size of a nut, of a very hard, gritty, and whitish substance.

This interesting case is, nevertheless, a very puzzling one; since no diagnosis of such fatal mischief could be had beforehand. The evil was too far gone to be remedied. Then why resort, it may be asked, to the painful and dangerous operation of gastrotomy? It is enough to reply that, when stercoral vomiting manifests an internal strangulation, *under such circumstances as these,* there is nothing *else* to be done; and from a mere artificial anus and peritoneal inflammation, some have recovered. *Gazette Méd. de Paris,* No. xli., 1838.

---

*On the Structure and Site of Condylomata.* By GUSTAV SIMON.

I. CONDYLOMATA, although usually divided into two varieties, the broad and pointed, are, anatomically considered, analogous in structure. When examined by the naked eye, they are seen to be composed of two substances: an external coat and an internal homogeneous mass. Microscopical examination shows that the external coat is a membrane nearly allied to the epithelium of mucous membranes; and that the internal mass is composed of numerous cells of various shapes, which, in the older part of the condyloma, are united into fibres, and these again into bundles. It has been shown by Schwann that all the animal tissues are, like those of vegetables, originally formed from cells, which are more or less modified, according to the stage of advancement at which the tissue has arrived; and Dr. Simon has found that the structure of cellular tissue, as described by Schwann, corresponds with that of the internal mass of condylomata.

*Müller's Archiv.* Heft i. 1839.

II. *Condylomata under the Tongue.* In a recruit to the fusilier battalion of the 31st Prussian infantry regiment, there were found condylomata under the tongue, which formed two rows of cockscombs, like lentils placed on their edges, extending on either side from the frænum to the point of the tongue. The patient's organs of generation were sound, and he affirmed that he never had chancre, but had suffered twice from gonorrhœa, two years ago. The patient was completely cured in four weeks by the internal use of sublimate, and by the external application of a solution of the same preparation, with extractum conii and honey.

*Militair-Medicinal Berichten.—Medicinische Zeitung. May* 15, 1839.

[*Query.* Was not this merely an enlargement of the crest or fold of the mucous membrane on the lower surface of the tongue, where the ducts of the sublingual glands open?]

---

*Case of Neuralgia, after Amputation, cured by Acupuncture.*
By FILIPPO LONGHI, M.D.

TERESA LONGHI (sister of the reporter), aged forty-nine, a Portuguese, had suffered amputation of the thigh for necrosis of the leg and white swelling of the knee-joint. A few months after the operation, severe pain, at irregular intervals, sometimes accompanied by fever, attacked the nerves of the stump. These pains were sudden and severe during the night, and appeared to the poor sufferer to recall her former agonies along the whole limb, previous to the amputation. Several external and internal remedies were tried without effect. At length, on the 19th of July, 1838, after six months' calm, she was again attacked by agonizing pains, not only in the stump, but extended to the opposite thigh, accompanied by convulsions and fainting, without any diminution of the suffering. In this state two needles were planted in the course of the sciatic nerve, from the nates downwards, towards the stump. These were productive of no effect: but scarcely had the third been introduced, when the patient exclaimed, "the doctor has struck the toe of my foot." Sr. Longhi then pressed the needle still deeper, so as to pass through the nerve, which was immediately followed by complete cessation of all pain. He then applied a fourth needle, without effecting any change. After the needles had been in half an hour, they were removed, and the patient enjoyed a profound sleep, and awoke free from pain. About twenty days after, the pain recurred, and the needles being again applied, again produced immediate relief.

*Bulletino delle Scienze Med. Ottobre,* 1838.

---

*On the Use of Acetate of Morphia in Arthritis, and Nervous Affections.*
By M. Vincent Cristin, Physician to the Hospital of St. John, Turin.

The following is Dr. Cristin's method of administering the acetate of morphia in these affections. Dissolve one grain of acetate of morphia in four ounces of distilled water, and add one ounce of syrup of gum arabic. A spoonful of this mixture to be taken every hour. When the pains are relieved, or sleep is about to commence, it should be given every two hours only, or suspended altogether; depending upon the narcotic effects produced. During its administration the patient should avoid fluids.

A woman, aged sixty, was afflicted with arthritis in the extremities; there was acute pain upon motion, hard pulse, burning heat of skin, &c. &c. The acetate of morphia relieved the pains, procured sleep, removed the fever, caused profuse perspiration and diarrhœa, with abundant secretion of urine. Opium excites perspiration, but diminishes the other secretions. Acetate of morphia is therefore the preferable agent.

Nervous pains, such as frontal neuralgia, sciatica, cephalalgia, and syphilitic pains, have all yielded to this remedy.

*Repertorio Medico-Chirurgico del Piemonte, Jan. and Feb.* 1838.

---

*Epilepsy treated with Belladonna.* By M. Jules Picard.

Since the 9th of September, 1837, twenty-two patients, afflicted with epilepsy, have been treated with belladonna in the wards of M. Ferrus. In six it produced symptoms which indicated the propriety of its discontinuance. In eight it was employed during a space of time, varying from forty days to four months and a half. In these it was discontinued, either on account of its producing no effect, or because the patients were tired of the treatment, or wished to leave the hospital. The remaining eight continued the treatment. Three commenced with 4 grains, fourteen with 6 grains, one with 9 grains, and three with 12 grains. The largest dose given was 18 grains. In three cases the epileptic seizures were not so frequent. *Revue Médicale, April,* 1838.

